# *Escherichia coli* O157:H7, a Common Contaminant of Raw Milk from Ecuador: Isolation and Molecular Identification

**DOI:** 10.3390/foods14030410

**Published:** 2025-01-27

**Authors:** Anthony Loor-Giler, Marcela Robayo-Chico, Byron Puga-Torres, Fernanda Hernandez-Alomia, Silvana Santander-Parra, Antonio Piantino Ferreira, Claire Muslin, Luis Nuñez

**Affiliations:** 1 Laboratorios de Investigación, Dirección General de Investigación, Universidad de las Américas (UDLA), Antigua Vía a Nayón S/N, Quito EC 170124, Ecuador; a.abel.loor.giler@gmail.com; 2 Facultad de Ingeniería y Ciencias Aplicadas, Carrera de Ingeniería en Biotecnología, Universidad de Las Américas (UDLA), Antigua Vía a Nayón S/N, Quito EC 170124, Ecuador; marcela2001alejandra@gmail.com; 3 Facultad de Medicina Veterinaria y Zootecnia, Universidad Central del Ecuador, Jerónimo Leyton s/n y Gilberto Gatto Sobral, Quito EC 170521, Ecuador; bpuga@uce.edu.ec; 4 Grupo de Investigación en Biodiversidad, Medio Ambiente y Salud (BIOMAS), Universidad de las Américas, Quito EC 170125, Ecuador; fgha97@hotmail.com; 5 Facultad de Ciencias de la Salud, Carrera de Medicina Veterinaria, Universidad de Las Américas, Antigua Vía a Nayon S/N, Quito EC 170124, Ecuador; silvanahsp@yahoo.com (S.S.-P.); claire.muslin@gmail.com (C.M.); 6 Laboratory of Avian Diseases, School of Veterinary Medicine and Animal Science, Department of Pathology, University of São Paulo, São Paulo 05508-270, SP, Brazil; ajpferr@usp.br; 7 One Health Research Group, Facultad de Ciencias de la Salud, Universidad de Las Americas, Quito EC 170124, Ecuador

**Keywords:** *E. coli* O157:H7, enterohaemorrhagic, raw milk, contamination, qPCR

## Abstract

*Escherichia coli* (*E. coli*), especially the Shiga toxin-producing O157:H7 strain, poses severe health risks. In rural Ecuador, raw milk consumption heightens contamination risks. This study analyzed 633 raw milk samples from Pichincha and Manabí to assess *E. coli* O157:H7 prevalence. The samples were enriched using BHI broth, and then specific culture media were used to isolate *E. coli* O157:H7. The pathogen in the enriched raw milk was identified, and the isolates were specifically confirmed through the application of a newly designed qPCR assay. The novel qPCR assay demonstrated remarkable sensitivity, capable of detecting up to one copy of genetic material, and specificity (no amplification of other bacteria). An extremely high *E. coli* O157:H7 prevalence of 0.63 (n = 401) was detected, where the province with the highest number of positive samples was Manabí with 72.8% (n = 225/309) and 54.3% (n = 179/324) for Pichincha. In both provinces, the presence of *E. coli* O157:H7 contamination exhibited a favorable correlation with small-scale farms and elevated temperatures. This research provides valuable data on the microbiological contamination of *E. coli* O157:H7 present in raw milk, in addition to an improved method that has been demonstrated to be faster, more sensitive, and more specific than conventional and previously published methods, highlighting the associated risk of food-borne infections and pointing out potential shortcomings in the regulation of agricultural practices and the need for periodic monitoring of bacterial contamination levels with updated methods.

## 1. Introduction

The dairy industry is a fundamental component of commerce and society, providing milk and dairy products that are in high demand among the population. Ecuador produces approximately 6.15 million liters of raw milk per day, according to the National Institute of Statistics and Census [1]. Dairy production represents a source of income for almost 1.2 million people. The dairy industry accounts for about 4% of the country’s agri-food GDP, with a large economic impact and a high export potential. According to data from the Internal Revenue Service (SRI), in September 2023, the dairy sector grew by 10.92% compared to the same month in 2022, being an expanding sector in response to population growth. The annual milk consumption in Ecuador is 110 L per person, according to data from the Ministry of Agriculture and Livestock (MAG), including raw milk (milk that has not undergone pasteurization) [2,3]. In Ecuador, there are approximately 299,000 milk producers, of which 80% belong to small farms and only 20% to medium and large farms. Only 4% of producers are technically trained and report high productivity [1].

Milk is the ideal culture medium for the proliferation of microorganisms [4] and may also contain antibacterial residues due to indiscriminate and irresponsible use of antimicrobials in dairy farming [5]. If the concentration of antimicrobials in milk exceeds the permitted limit stipulated by AGROCALIDAD [6], the main risk associated with its consumption is the generation of antimicrobial resistance in the human host’s microbiota or even pathogenic bacteria present in the organism and in the milk [5,7]. A high variety of microorganisms have been previously reported as prevalent in raw milk [8], specifically the genera *Staphylococcus* [9], *Streptococcus* [10], *Acinetobacter* [11], *Salmonella* [12], *Campylobacter* [13], *Listeria* [14], and *Escherichia* [15]. Within the *Escherichia* genus, *Escherichia coli* (*E. coli*) is the most commonly reported bacteria.

While many *E. coli* strains are non-pathogenic [16], enterohaemorrhagic *E. coli* (EHEC) has been identified as one of the most virulent groups due to its ability to produce the Shiga toxin. This toxin exerts its effect by binding to the 60S ribosomal subunit in intestinal or renal cells, with pathogenicity influenced by the presence of STX1 and STX2 variants [17]. The serovar *E. coli* O157:H7 is the most notable within this group, frequently associated with outbreaks linked to contaminated meat and dairy products. Clinical symptoms often include bloody diarrhea, hemolytic uremic syndrome, and thrombocytopenia, conditions closely related to microangiopathic hemolytic anemia [18].

*E. coli* O157:H7 has caused major outbreaks worldwide, including a 2006 spinach-related outbreak in the United States of America (USA) (205 cases, 3 deaths) [19], a 2018 romaine lettuce outbreak (210 cases, 5 deaths) [20], and a 2011 soybean sprout outbreak in Germany (over 50 deaths) [21]. These incidents emphasize the need for strict food safety measures to prevent infections, as highlighted by the World Health Organization (WHO), particularly due to risks like hemolytic uremic syndrome in vulnerable populations [22]. Although specific reports of *Escherichia coli* O157:H7 contamination in milk and dairy products within Ecuador are limited, this pathogen has been identified in Ecuadorian livestock, indicating potential animal reservoirs [23]. In the Central Highlands of Peru, *E. coli* O157:H7 has been isolated from beef samples and dairy calves [24]. In neighboring Colombia, the first documented human infection with *E. coli* O157:H7 was reported in 1998, underscoring the pathogen’s presence in the region [25]. Although no pathogen-associated illnesses have been identified in Ecuador to date, this may be a bias associated with an inadequate form of diagnosis; the absence of accurate diagnostic methods, common in developing countries, can make it very difficult to accurately associate *Escherichia coli* O157:H7 with disease outbreaks and related deaths [20,23,26].

Dairy production in Ecuador is mainly informal, so quality control methods are scarce and inefficient [27]; consequently, good animal husbandry practices are in decline, increasing the rates of bacterial contamination [6]. Accurate and specific detection of *Escherichia coli* O157:H7 in raw milk remains a challenge due to the complex nature of this matrix and the potential for cross-reactivity with other *E. coli* serovars in the case of serological analysis. Traditional culture techniques are time-consuming and may not differentiate pathogenic from non-pathogenic strains, while existing molecular methods often lack the specificity necessary for reliable identification of this strain of *E. coli* as they possess low standards of sensitivity and specificity.

The main objective of this study is to isolate and perform molecular identification of *E. coli* O157:H7 in raw milk samples from Ecuador, especially from the provinces of Pichincha and Manabí, to identify specific factors contributing to elevated contamination rates, such as the weather season (because temperature changes can influence the proliferation of certain bacteria) or from small and medium-sized producers (who manage different biosecurity standards). The use of microbiological and molecular tools such as a new qPCR will allow for the specific detection of this bacteria, taking into account the need for continuous updating of diagnostic procedures, as well as the need for faster, more sensitive, and more specific methods, based on updated records of genes associated with this pathogen, such as the fimbrial gene *z3276* [28]. Additionally, a comprehensive statistical analysis of all collected results will be performed to assess the prevalence and distribution of *E. coli* O157:H7 in raw milk samples and to identify any significant correlations with parameters such as geographic location or production conditions. This integrated approach that utilizes microbiological, molecular, and statistical tools will allow us to gain a comprehensive understanding of the presence of these pathogenic bacteria in raw milk from Ecuador, serving as a catalyst for the enforcement of stricter laws on culturally consumed foods.

## 2. Materials and Methods

### 2.1. Sample Collection

A total of 633 samples were collected from two provinces of Ecuador: Pichincha (324 samples) and Manabí (309 samples). The samples collected corresponded to milk intended for human consumption from cows with no apparent mastitis. This process was carried out with the collaboration of students from the Universidad Central del Ecuador (UCE), who took raw milk samples from small (527 samples) and medium (106 samples) producers from 5 September 2022 to 16 July 2023 tanking considering rainy (457 samples) and warm (176 samples) conditions. The collection of these samples was carried out based on NTE INEN ISO 707 and Standard ISO 7218, under aseptic conditions, keeping the samples in sterile containers and with adequate temperature settings (4 °C) until they were received at the research laboratories of the Universidad de Las Americas (UDLA). The collection also followed parameters to classify the producers as small and medium-sized. According to Ministerial Agreement No. 095, small producers are defined as those who have between one and fifty head of cattle, while medium-sized producers have between fifty and two hundred head of cattle [29]. The climatic conditions indicated were made according to the National Institute of Meteorology and Hydrology (INAMHI), which indicates a warm or dry season from June to September with high temperatures (between 20 °C and 30 °C) and little rainfall and a rainy or wet season from October to May with lower temperatures (between 18 °C and 22 °C) and constant rainfall. All procedures conducted in the present investigation were in line with the guidelines and the approval of the Committee on the Care and Use of Laboratory and Domestic Animal Resources of the Agency of Regulation and Control of Phytosanitary and Animal Health of Ecuador (AGROCALIDAD), under number #INT/DA/019.

#### Sample Size Determination

The number of samples needed for this study was calculated, determining an expected prevalence of *E. coli* O157:H7 of 50% in the study, a desired absolute precision of 5%, and a confidence level of 95%, using the formula $n=\frac{Z^{2}*P_{exp}(1-P_{exp})}{d^{2}}$, where n = required sample size; Pexp = expected prevalence; d = desired absolute precision; z = statistic (standard normal distribution) for a level of confidence of 95% = 1.96. Thus, the minimum number of raw milk samples required to determine the prevalence of *E. coli* was calculated to be 384 milk samples [30].

### 2.2. Primers Design

In this study, we utilized previously published primers and hydrolysis probes for the molecular identification of *E. coli*, centered on the *lacY* gene, which encodes the lactose permease [31]. Specific primers and hydrolysis probes for molecular detection of *E. coli* O157:H7 were designed to target a conserved part of the open reading frame z3276, denominated as a *fimbrial* gene related to bacterial pathogenicity, which is a high specific genetic marker of *E. coli* O157:H7 [28,32] (Table 1). Primer design was carried out using the Geneious Prime 2022.1.1 (https://www.geneious.com) software package [33]. An alignment was performed using Clustal X v1.83, utilizing multiple reference sequences obtained from NCBI, and the most similar regions among them were identified. The analysis of the non-prevalence of potential non-specific hybridizations was conducted using the BLASTn tool.

### 2.3. Standard Curve Construction and Sensitivity Assay

A synthetic double-stranded DNA fragment (gBlocks Gene Fragments, IDT) containing the target genomic sequences target for primers to identify *E. coli* and *E. coli* O157:H7 was in silico designed and obtained from Integrated DNA Technologies (IDT) for standard curve formulation [34]. The engineered DNA fragment was dissolved in 50 μL of UltraPure™ DNase/RNase-Free Distilled Water (Invitrogen) following the manufacturer’s instructions. This solution was quantified on a NanoDrop™ 2000 instrument (Thermo Fisher Scientific). The DNA concentration obtained was entered into the DNA Copy Number and Dilution Calculator well tool (Thermo Fisher Scientific) to determine the amount of DNA fragment needed to generate a standard dilution with 10^8^ copies of genetic material per µL. Finally, serial one-tenth dilutions of the DNA standards were performed until 1 copy of genetic material was reached for the construction of standard curves for qPCR assay and to determine the sensitivity of the assay.

### 2.4. Analysis of Specificity

The new detection method for *E. coli* O157:H7 based on a qPCR assay was tested with extracted DNA corresponding to positive controls of the strains *Escherichia coli* ATCC 25922, *Klebsiella pneumoniae* ATCC 13883, *Salmonella typhimurium* ATCC 14028, *Shigella flexneri* ATCC 12022, and *Escherichia coli* O157:H7 ATCC 35150.

### 2.5. Milk Enrichment

The samples were subjected to an enrichment process with BHI broth according to the previously described protocol [35] in a ratio of 90% medium and 10% milk in a total of 40 mL. These samples were incubated at 37 °C for approximately 24 h with 150 g agitation.

#### DNA Extraction for Enriched Milk

Bacterial DNA from enriched milk was extracted to determine the presence or absence of *E. coli* O157H7 genetic material in it, performed through qPCR. In 1.5 mL tubes, 1 mL of enriched milk sample was placed and stored at −20 °C for approximately 24 h. The tubes were then heated in a 56 °C water bath and centrifuged at 12,000× *g* for 10 min. After that, an 800 µL aliquot of the supernatant was taken and used for DNA extraction with phenol/chloroform, as outlined in the previously described protocol [36].

### 2.6. Bacteria Isolation

All enriched samples were subjected to an isolation process on MacConkey Agar with Sorbitol, a selective medium for *E. coli* 0157:H7, following the guidelines of the International Standard ISO 16654:2002/A2:2023 [37]. Following this, the isolated colonies were identified according to their phenotype. On selective MacConkey Agar with Sorbitol, *E. coli* O157:H7, which does not ferment sorbitol, forming transparent colonies, whereas most *E. coli* strains ferment sorbitol, appearing as pinkish-reddish colonies.

#### DNA Extraction for Isolated Bacteria

DNA was extracted from isolated colonies with phenotypic characteristics of *E. coli* O157:H7 to genetically confirm the isolation of this bacteria through molecular assays. The colonies were selected and placed in 1.5 mL tubes with BHI broth. These tubes were centrifuged at 12,000× *g* for 10 min at 4 °C to pellet the cells, the supernatant was discarded, and 200 µL of TE buffer was added into each tube, homogenized with vortex, and stored at −20 °C for approximately 24 h. After approximately 24 h, the tubes were warmed at 95 °C in a dry bath (Thermo Fisher Scientific, Carlsbad, CA, USA) and gently homogenized. The samples were then centrifuged at 12,000× *g* for 10 min at 4 °C, and finally, 100 µL of the supernatant was aliquoted into 200 µL microtubes. The extracted DNA was stored at −20 °C until use.

### 2.7. Molecular Detection of Escherichia coli O157:H7 Using qPCR

For bacteria identification, duplex qPCR reactions were performed, using a 10 µL MasterMix consisting of 2X TaqMan™ Universal Master Mix II, with UNG (Thermo Fisher Scientific, Carlsbad, CA, USA), 0.2 µM of each primer, 0.1 µM of hydrolysis probe (Table 1), and 1 µL of DNA diluted 1:5. The amplification by qPCR was carried out under the following conditions: 1 cycle at 50 °C for 2 min for the inactivation of UNG, 1 cycle at 95 °C for 5 min for the initial denaturation followed by 45 cycles at 95 °C for 15 s during denaturation, 60 °C for 45 s for reading and annealing, and 72 °C for 30 s for extension. For this, the CFX96 Touch Real-Time PCR Detection System thermal cycler (Bio Rad Laboratories, Inc., Hercules, CA 94547, USA) was used. These qPCR protocols were used to detect the presence of *E. coli* O157:H7 in the DNA extracted from enriched raw milk and to confirm the bacterial isolates with selective media. In the analysis of detection data concerning enriched milk and isolates, samples that amplified for both targets were classified as *E. coli* O157:H7, confirming the serotype. Conversely, those that amplified solely for *E. coli* were categorized as non-O157:H7 *E. coli*.

### 2.8. 16S Sequencing and Bioinformatic Analysis

Using extracted DNA from *E. coli* O157:H7 isolates, 16S rDNA (approximately 1400 bp) was amplified with universal primers 27F/1492R (Table 1) [38], using GoTaq Green Master Mix (Promega, Madison, WI, USA). PCR conditions included 40 cycles of 95 °C, 55 °C, and 72 °C, followed by gel electrophoresis with SYBR Safe staining (Thermo Fisher Scientific, Carlsbad, CA, USA). Amplicons were purified using ExoSAP-IT™ (Applied Biosystems, Santa Clara, CA 95051, USA), sequenced bidirectionally using BigDye^®^ Terminator v3.1 (Applied Biosystems, Santa Clara, CA, USA), and analyzed on an ABI 3500 Genetic Analyzer (Applied Biosystems, Carlsbad, CA, USA). The electropherograms generated were aligned using a reference sequence of each analyzed bacteria in Geneious software version 10.2.3 (https://www.geneious.com) [33] and analyzed by BLAST and compared to other *E. coli* O157:H7 sequences deposited in GenBank. The aligned sequences were used for the construction of a nucleotide similarity matrix with the help of Geneious software version 10.2.3 (https://www.geneious.com) (Biomatters Ltd., Auckland, New Zealand).

### 2.9. Multilocus Typing Sequencing (MLSTs) for Strains Identification

Fifteen DNA samples of *E. coli* O157:H7 isolates were randomly selected and subjected to the MLST identification previously described protocol [39]. The 7 strands (gyrB, mdh, recA, icd, fumC, adk, purA) per sample were amplified using 2X Promega GoTaqTM G2 Green Master Mix enzyme and sequenced using the BigDye^®^ Terminator v3.1 Cycle Sequencing Kit (Thermo Fisher Scientific, Carlsbad, CA, USA) according to the supplier’s instructions. The results were delimited based on the reference sequences present on the PubMLST website and subjected to PubMLST to identify the allelic profile of each strain. The results were used to reconstruct a minimum spanning tree (MSTs) using PHYLOViz 2.0 online software [https://online.phyloviz.net/index (accessed on 25 November 2024)] and the goeBURST algorithm.

### 2.10. Statistical Analysis

qPCR results were organized using descriptive statistics based on province and producer size. The data were then imported into the R Studio 2022.12.0 + 353 environment (R Core Team, 2021). The prevalence of *E. coli* O157:H7 in raw milk from Ecuador was calculated using the data from the positive samples for isolation of the strains validated with the qPCR assay, based on the formula: $Prevalence=\frac{\#\;of\;members\;in\;sample\;with\;characteristic}{Total\;\#\;of\;members\;in\;sample}$, as described on the NCBI website [https://www.nimh.nih.gov/health/statistics/what-is-prevalence#:~:text=For%20a%20representative%20sample%2C%20prevalence,of%20people%20in%20the%20sample (accessed on 20 November 2024)]. To carry out the statistical tests, distribution tests were performed; first a Shapiro–Wilk test was performed to determine whether the data loaded into the environment follow a normal distribution to run multivariate tests. For this analysis, the chi-square test was applied to analyze the association between variables (province, climate, and producer size). The confidence level for these tests was 95%.

## 3. Results

### 3.1. Sensitivity Assay

The standard curve allowed the determination of LoD (limit of detection) and LoQ (limit of quantification), showing that these values were up to 1 gene copy/µL. For the *E. coli* O157:H7 qPCR assay, the standard curve generated an efficiency of 96.0%, a slope of −3.421, and a correlation coefficient of 0.998 (Supplementary Material).

### 3.2. Specificity Assay

Of the positive controls used (*Escherichia coli* ATCC 25922, *Klebsiella pneumoniae* ATCC 13883, *Salmonella typhimurium* ATCC 14028, *Shigella flexneri* ATCC 12022, and *Escherichia coli* O157:H7 ATCC 35150), only the one corresponding to the *E. coli* O157:H7 strain showed amplification, showing the specificity of the method against the bacteria corresponding to these strains.

### 3.3. qPCR Detection in Enriched Milk

In total, out of the 633 samples analyzed, 401 (63.4%) were positive for *E. coli* O157:H7, and 121 (19.11%) were positive for non-O157:H7 *E. coli* (Table 2). The frequency of *E. coli* O157:H7 in raw milk samples was higher in Manabí than in Pichincha, with 225 (72.8%) compared to 176 (54.3%) with significative differences. The presence of *E. coli* O157:H7 in raw milk was significantly higher in samples from small producers located in warm climate areas in the province of Manabí compared to the other groups. Additionally, small producers showed a higher contamination rate for *E. coli* O157:H7 and non-O157:H7 in both provinces compared to medium producers, with 151 and 186 positive samples for Pichincha and Manabí, respectively. Positive samples for non-O157:H7 *E. coli* were higher in Pichincha (n = 86) than in Manabí (35) without showing significant differences in any of the analyzed groups based on climate and producer size. Considering the presence of any strain of *E. coli*, 522 positive samples were identified (82.46%), indicating a high prevalence in the country.

### 3.4. Escherichia coli O157:H7 Isolation and qPCR Validation of Strains

A concordance of 99.75% was observed between the *E. coli* O157:H7 detection results from bacterial isolation on selective media and those from the qPCR assay conducted directly on enriched raw milk samples (Table 2), with only one additional sample testing positive in the qPCR assay and a Kappa coefficient > 0.9, indicating a high degree of similarity in the results. These results showed that the prevalence of *E. coli* O157H7 was 0.63. For the samples positive for non-O157:H7 *E. coli*, 100% concordance was obtained in both results.

### 3.5. 16S Analysis

The nucleotide similarity matrix between the *E. coli* O157:H7 sequences obtained in this study and sequences randomly collected from NCBI reveals high similarity, with percentages ranging from 97.65% to 99.963%. The sequences obtained in the study have outstanding nucleotide similarities with sequences from various parts of the world, including the United States of America (USA) (AY513502.1), Germany (HM007589.1), Vietnam (HQ658163.1), India (KX984112.1), Iraq (OM442028.1), and Egypt (MT705744.1). These similarities suggest a close genetic relationship between local and international strains, indicating a global distribution and circulation and possible common evolution of these pathogens. In particular, the sequence “LM 92” shows a 99.367% similarity to the USA sequence AY513502.1, and “LM 196” has a 99.478% similarity to the Egyptian MT705744.1 sequence (Table 3).

### 3.6. MLSTs

A total of 15 *E. coli* O157:H7 isolates subjected to analysis revealed an extensive range of sequence types (STs). A predominance of the tested samples, four each, were associated with the ST11 complex and the ST10 complex. One sample (86LM) was confirmed to belong to the ST206 complex, and finally, six samples showed no relationship to any complex previously reported in the PupMLST database (Table 4).

**Figure 1:**
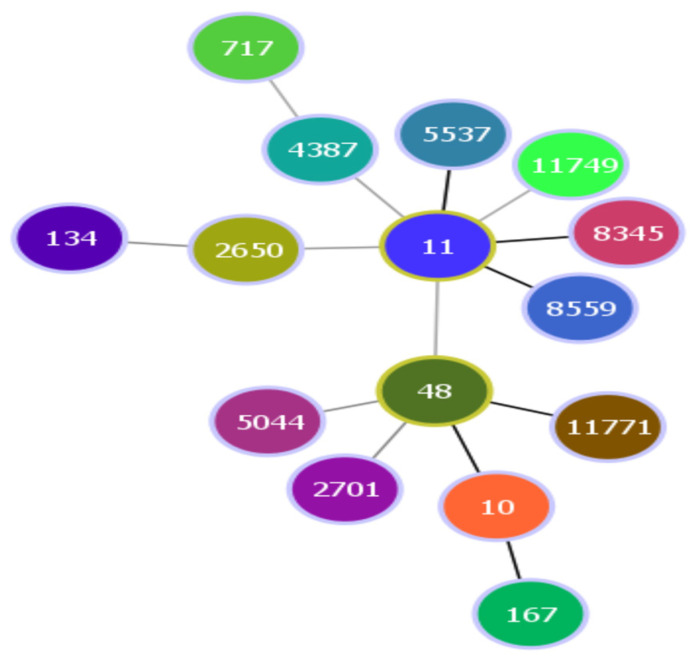
Minimum spanning tree (MST) generated using the PHYLOViz tool from the allelic profiles of 15 *E. coli* O157:H7 isolates from raw milk from Ecuador. Each ST is represented by a circle. The length of the lines between each ST shows proportionally the number of different alleles. The strain types connected with gray lines are more distantly related to the central nodes.

Strains within the complex ST10 (ST10, 48, 167, and 11771) and complex ST11 (ST11, 5537, 8345, and 8559) were more common. It is noteworthy that ST11 and ST48 were identified as central nodes within the minimum spanning tree (MST). ST11 demonstrated a strong relationship with ST5537, 8345, and 8559, while ST48 exhibited a strong relationship with ST10 and ST11771 (Figure 1).

## 4. Discussion

Milk and dairy products are highly susceptible to contamination by various chemical and biological pathogens [3,8]. Among these, biological agents, mainly bacteria, are the most common, although viruses and parasites can also be found [8]. The present study showed the presence of *E. coli* O157:H7 in raw milk in Ecuador, presenting an evident risk to public health. Our newly designed qPCR method used for the identification of this bacterium showed high similarity with the bacterial isolation methods (Table 2), indicating that the direct detection of *E. coli* O157:H7 from the DNA of enriched milk is a fast, sensitive, and adequate alternative for the diagnosis of this pathogen. Several studies have used mass sequencing as a mechanism to detect and report the presence of *E. coli* O157:H7 along with a wide range of other bacteria [40,41]. However, these methods are very costly and inaccessible to small and medium-sized agricultural testing laboratories; hence, rapid and economic methods such as qPCR are presented as an alternative for diagnosis [18,42]. Previous qPCR-based methods have shown an inability to detect up to one copy of bacterial genetic material [43,44], in addition to specificity problems in the presence of other enterobacteria. Therefore, the present study is shown as a viable alternative for detection in environments with high levels of contamination, such as raw milk. Furthermore, it is necessary to update diagnostic methods based on the constant new publications and additions of sequences to the databases, in particular of the *Z3276* gene recently linked to the pathogenic effect of this *E. coli* serovar [28].

*E. coli* O157:H7 was detected in 63.4% (401/633) of the raw milk samples analyzed from the provinces of Pichincha and Manabí in Ecuador. These results are of great concern for public health due to the high production and consumption of raw milk daily in Ecuador [1]. A significant portion, ranging from 50 to 70%, is distributed through informal channels, primarily as liquid raw milk or artisanal dairy products like fresh cheese. These products often do not undergo sufficient thermal treatment to eliminate pathogenic microorganisms, thereby presenting a potential risk to public health in Ecuador [45,46,47]. Since its discovery as a pathogen, *E. coli* O157:H7 has been regularly reported in the intestines of cattle [48], indicating its presence in their feces. This bacterium can be transmitted through contaminated drinking water or various foods, including raw milk. Such pathways may contribute to significant contamination levels, compounded by inadequate management practices among farmers, which have greatly facilitated the proliferation of this pathogen that remains insufficiently regulated [49].

The qPCR assay for the detection of this pathogen in enriched raw milk showed that both Manabí and Pichincha have a higher presence in small producers (Table 2), attributed to a report by AGROCALIDAD [50], which indicates that manual milking is the predominant practice among small dairy farmers, prompting the regulatory body to include quality and biosecurity guidelines in its annex “Guide to Good Livestock Practices in Dairy Farming” [6]. However, previous studies mention that small producers who carry out manual milking do not comply with the established standards [51], which implies that raw milk and dairy products contain high levels of microbiological contamination.

The highest incidence of bacterial contamination was found in samples from Manabí (72.8%, n = 225). *E. coli* O157:H7 is a mesophilic bacterium, which grows at moderate temperatures, typically between 20 °C and 45 °C. The environmental conditions of the coastal region, where Manabí is located, create an environment conducive to microbial contamination [52], driving the proliferation of this and other contaminating microorganisms. Previous research has shown that these conditions significantly increase the risk of bacterial contamination in raw milk compared to industries with better equipment and regulations [45]. Environmental and socio-economic conditions in Pichincha are slightly better, with a more developed infrastructure to maintain the necessary hygiene standards [53]. Nevertheless, important challenges remain, considering that the presence of the single pathogen identified in this study was found in 54.32% of the samples analyzed (Table 2).

*E. coli* O157:H7 may even have the ability to survive at a potentially sub-lethal temperature for long periods of time [54] and may, therefore, be present in dairy products that have not received adequate heat treatment. Outbreaks of food-borne illnesses caused by *E. coli* O157:H7 have been documented worldwide. In England (2019), 57% (12/21) of cases required hospitalization for hemolytic uremic syndrome [39]. In Canada (2013), Gouda cheese made from raw milk contaminated with *E. coli* O157:H7 caused illness in 29 individuals, including five emergency hospitalizations and one fatality [46]. Similarly, in Ogun, Nigeria, 5% (10/202) of tested samples were positive for *E. coli* O157:H7, including raw milk, locally produced fresh and fried cheeses, and fermented milk [54]. In Ecuador, the Ministry of Public Health (MSP) reported 5872 cases of food-borne infections in 2021 without a confirmed diagnosis of the causative pathogen [55]. Considering the high prevalence of *E. coli* O157:H7 in this study, there is an emerging risk of food-borne diseases associated with non-regulated foods in the country.

When analyzing the 16S sequences of the strains isolated in the present study, a high similarity with those previously deposited in GenBank was observed (Table 4), proving the phylogenetic relationship of these strains. Allelic profiling with MLSTs showed 15 different STs (Figure 1), 9 of them related to complexes described as hyperpathogenic [56]. In particular, the ST206Cplx complex has been associated with beta-lactamase-associated antimicrobial resistance, as well as resistance to tetracyclines, sulfonamides, trimethoprim, and aminoglycosides [57,58,59,60]. Conversely, the ST10Cplx complex has been linked to the production of the Shiga toxin as a result of the effect of *stx1* and *stx2* genes present in several *E. coli* EHEC serovars, including O157:H7 and O104:H4. This complex has been primarily isolated from agricultural environments, particularly in poultry farming [57]. On the other hand, the ST11Cplx complex has been described as the distinctive ST of serovar O157:H7 [48,61] and is usually found in food for human consumption [62]. The evidence on intrinsic antimicrobial resistance in this ST is unclear; while some articles highlight multi-resistant strains [63], others point out that this group has a low number of resistance genes [64]. Therefore, it is necessary to perform local studies to identify the risk of antimicrobial ineffectiveness. On the other hand, 6 samples were not related to any complex (Table 4). Individually, they have been reported to be present in different environments, such as fermented foods, milk derivatives, meat, and pickles [65,66]. Even though some of these STs, such as ST134, have been related to some resistance genes like beta-lactamases (among others), there is not enough information about them; consequently, further studies are needed to analyze their complete genomes and define their pathogenic potential [66]. The MLST data of the *E. coli* O157:H7 strains collected in this study are not sufficient to determine the possible source of contamination or problems associated with the totality of specific strains present in raw milk. Future, more comprehensive studies that analyze a larger and more significant number of samples both in MLSTs and in other molecular analysis methods such as Whole Genome Sequencing (WSG) are needed [40].

Several countries such as the USA, Germany, China, Nigeria, Canada, Netherlands, and Paraguay, among others [5,20,40,46,67,68,69], have had public health emergencies due to food contamination by *E. coli* and other pathogens such as *Salmonella*, *Klebsiella*, *Staphylococcus*, *Listeria*, *Campylobacter*, *Pseudomonas*, and *Acinetobacter*, among others, which have endangered public health. For this reason, and taking into account the circulating strains evidenced in this study, it is imperative to conduct additional research to determine the severity of the contamination issue in food (massive detection), take into account other potential microorganisms, determine the extent to which it is impacting public health (epidemiological analysis), and, finally, to determine the phenomena of bacterial resistance found in all of these. This can be achieved through the use of qPCR methods for primary diagnostics and investigation or cutting-edge applications such as NGS (Next Generation Sequencing). Many factors may influence the increased rates of bacterial contamination in raw milk in addition to those examined in this study, such as individual good animal husbandry practices, the use of certain containers, or the proper decontamination of all equipment used. It is necessary for producers to follow the guidelines of good animal husbandry practices and to carry out periodic quality studies [70,71]. Future studies are needed to take these factors into consideration in order to determine if any of these factors served as a bias for this study, thus increasing the contamination rate of the pathogen identified here.

## 5. Conclusions

The findings of this study underscore the significant public health risks associated with the contamination of milk and dairy products by *E. coli* O157:H7 in Ecuador. The high prevalence of this pathogen, particularly in raw milk from Manabí and Pichincha, highlights the urgent need for improved regulatory frameworks and adherence to biosecurity practices, especially among small producers. The qPCR method developed in this study proved to be a rapid, sensitive, and cost-effective diagnostic tool, demonstrating its potential as a practical alternative to traditional and high-cost sequencing methods for pathogen detection. Furthermore, the phylogenetic analysis revealed hyperpathogenic strain complexes and antimicrobial resistance profiles, emphasizing the necessity of ongoing genomic surveillance to monitor and mitigate the risk of emerging multi-drug-resistant strains. Given the global and local burden of food-borne illnesses caused by *E. coli* O157:H7, the implementation of advanced diagnostic methods, together with stricter food safety measures, is critical to safeguard public health.

## Figures and Tables

**Table 1:** Primers and probes used in this study.

Name	Target	Sequence	Reference
**EClpma (−1)**	*lacY* gene	5′-ACCAGACCCAGCACCAGATAAG-3′	[31]
**lacY_t_rev1**		5′-CTGCTTCTTTAAGCAACTGGCGA-3′	
**lacY_p1**		FAM/5′-CATACATATTGCCCGCCAGTACAGAC-3′/1BHQ	
***E. coli* 0157:H7-F**	z3276 locus	5′-TGCGTGGTAAAACAGATAATGGTG-3′	This study [AE005174]
***E. coli* 0157:H7-R**		5′-TGTTTTCCATAATGATGTCGC-3′	
***E. coli* 0157:H7-P**		CY5/5′-ACAGAACCACCAAAAGGGACTGAGGCTG-3′/3BHQ	

**Table 2:** Analysis of bacteria isolation in selective media and qPCR detection in enriched milk for the two bacteria detected in relation to the locality of the samples, producer size, and percentage for each bacterium of positive samples over the total number of samples in the province.

Province	Producer Size	Climate/Collection Date	*Escherichia coli* (non-O157:H7)	*Escherichia coli* O157:H7	Negative Samples	Total of Samples
**Pichincha**	Small *	**Warm/Jun–Sep**	△38 (22%)	△85 (49%)	49	172
		**Rainy/Oct–May**	△30 (30.6%)	△66 (67.3%)	2	98
	**Medium**	**Warm/Jun–Sep**	△9 (36.5%)	◍14 (41.2%)/13 (38.2%)	11	34
		**Rainy/Oct–May**	△9 (45%)	△11 (55%)	0	20
Manabí *	Small *	**Warm/Jun–Sep**	△29 (13.8%)	△142 (67.6%) *	39	210
		**Rainy/Oct–May**	△1 (2.1%)	△44 (93.6%)	2	47
	**Medium**	**Warm/Jun–Sep**	△5 (12.2%)	△28 (68.3%)	8	41
		**Rainy/Oct–May**	△0 (0.0%)	△11 (100%)	0	11
		**Total of positives**	121 (19.1%)	401 (63.3%)	111	633

* Significant differences in statistical analysis between different microorganisms. △ Identical results in isolation (validated by specific qPCR) and molecular identification in enriched milk by qPCR. ◍ Non-identical results in isolation and qPCR assays divided in: left for qPCR and right for isolation. Jun–Sep = from June to September. Oct–May = from October to May.

**Table 3:** Comparison between the nucleotides of *Escherichia coli* O157:H7 sequences obtained in this study and the sequences collected from NCBI. Sequences obtained in this study are marked in light blue.

Sample ID	1.	2.	3.	4.	5.	6.	7.	8.	9.	10.	11.	12.	13.	14.	15.	16.
1. AY513502 USA	-	99.926	99.242	98.951	99.408	98.975	99.255	99.367	98.176	98.362	98.436	98.660	99.330	98.362	99.479	99.032
2. HM007589 GERMANY	99.926	-	99.335	98.867	99.387	98.939	99.231	99.346	98.115	98.308	98.385	98.615	99.308	98.308	99.462	99.000
3. HQ658163 VIETNAM	99.242	99.335	-	97.830	98.890	98.553	98.883	98.846	97.655	97.915	97.915	98.138	98.809	97.915	98.958	98.585
4. KX984112 INDIA	98.951	98.867	97.830	-	99.071	98.683	98.921	99.033	97.842	97.954	98.028	98.251	98.903	97.954	99.070	98.698
5. OM442028 IRAQ	99.408	99.387	98.890	99.071	-	99.646	99.851	99.963	98.696	98.733	98.808	99.106	99.777	98.808	99.851	99.627
6. MT705744 EGYPT	98.975	98.939	98.553	98.683	99.646	-	99.644	99.728	98.388	98.388	98.388	98.723	99.561	98.472	99.560	99.477
7. LM 89 ECUADOR	99.255	99.231	98.883	98.921	99.851	99.644	-	99.814	98.547	98.584	98.659	98.957	99.628	98.659	99.702	99.478
8. LM 92 ECUADOR	99.367	99.346	98.846	99.033	99.963	99.728	99.814	-	98.659	98.696	98.770	99.069	99.739	98.770	99.814	99.590
9. LM 94 ECUADOR	98.176	98.115	97.655	97.842	98.696	98.388	98.547	98.659	-	98.845	99.218	98.696	98.511	98.696	98.621	98.323
10. LM 96 ECUADOR	98.362	98.308	97.915	97.954	98.733	98.388	98.584	98.696	98.845	-	98.733	98.882	98.511	99.478	98.882	98.510
11. LM 98 ECUADOR	98.436	98.385	97.915	98.028	98.808	98.388	98.659	98.770	99.218	98.733	-	98.957	98.585	98.510	98.957	98.584
12. LM 100 ECUADOR	98.660	98.615	98.138	98.251	99.106	98.723	98.957	99.069	98.696	98.882	98.957	-	98.883	99.255	99.106	98.733
13. LM 101 ECUADOR	99.330	99.308	98.809	98.903	99.777	99.561	99.628	99.739	98.511	98.511	98.585	98.883	-	98.585	99.628	99.404
14. LM 169 ECUADOR	98.362	98.308	97.915	97.954	98.808	98.472	98.659	98.770	98.696	99.478	98.510	99.255	98.585	-	98.808	98.584
15. LM 196 ECUADOR	99.479	99.462	98.958	99.070	99.851	99.560	99.702	99.814	98.621	98.882	98.957	99.106	99.628	98.808	-	99.478
16. LM 201 ECUADOR	99.032	99.000	98.585	98.698	99.627	99.477	99.478	99.590	98.323	98.510	98.584	98.733	99.404	98.584	99.478	-

**Table 4:** Allelic profile of the 15 *E. coli* O157:H7 samples analyzed in this study based on the 7 markers (gyrB, mdh, recA, icd, fumC, adk, and purA). STs and allelic complexes determined on the basis of PubMLST. Non-determined (ND).

Sample	N° of Alleles							ST	Complex
	gyrB	mdh	recA	icd	fumC	adk	purA		
**86 LM**	4	8	2	1	7	6	251	2701	ST206 Cplx
**89 LM**	49	16	34	13	44	13	10	134	ND
**137 LM**	155	24	42	322	209	12	2	4387	ND
**139 LM**	8	16	34	13	12	12	10	2650	ND
**143 LM**	8	15	2	12	12	12	2	11	ST11 Cplx
**260 LM**	4	7	2	109	11	57	8	5044	ND
**261 LM**	4	8	2	8	11	10	13	167	ST10 Cplx
**277 LM**	4	8	2	8	11	10	8	10	ST10 Cplx
**480 LM**	75	27	42	141	161	127	1	717	ND
**483 LM**	1	8	2	8	11	6	8	11771	ST10 Cplx
**484 LM**	4	8	2	8	11	6	8	48	ST10 Cplx
**501 LM**	41	5	2	43	45	20	957	11749	ND
**505 LM**	8	15	2	12	1153	12	2	8345	ST11 Cplx
**540 LM**	8	15	2	12	1182	12	2	8559	ST11 Cplx
**562 LM**	8	15	2	12	698	12	2	5537	ST11 Cplx

## Data Availability

The original contributions presented in the study are included in the article/Supplementary Materials, further inquiries can be directed to the corresponding author.

## Supplementary Materials

The following supporting information can be downloaded at https://www.mdpi.com/article/10.3390/foods14030410/s1. Figure S1: Standard curve for the qPCR assay for the detection of *E. coli* and *E. coli* O157:H7; Figure S2: Amplification plot for the standard curve of *E. coli* qPCR assay; Figure S3: Amplification plot for standard curve of *E. coli* O157:H7 qPCR assay.
