# Supplementary material for: Escherichia coli O157:H7, a Common Contaminant of Raw Milk from Ecuador: Isolation and Molecular Identification
Source: Foods. 2025 Jan 27;14(3):410. doi: 10.3390/foods14030410 (PMC11816838; doi:10.3390/foods14030410)
Supplement: Supplementary file 1 [file foods-14-00410-s001.zip › foods-3403066-supplementary.pdf]

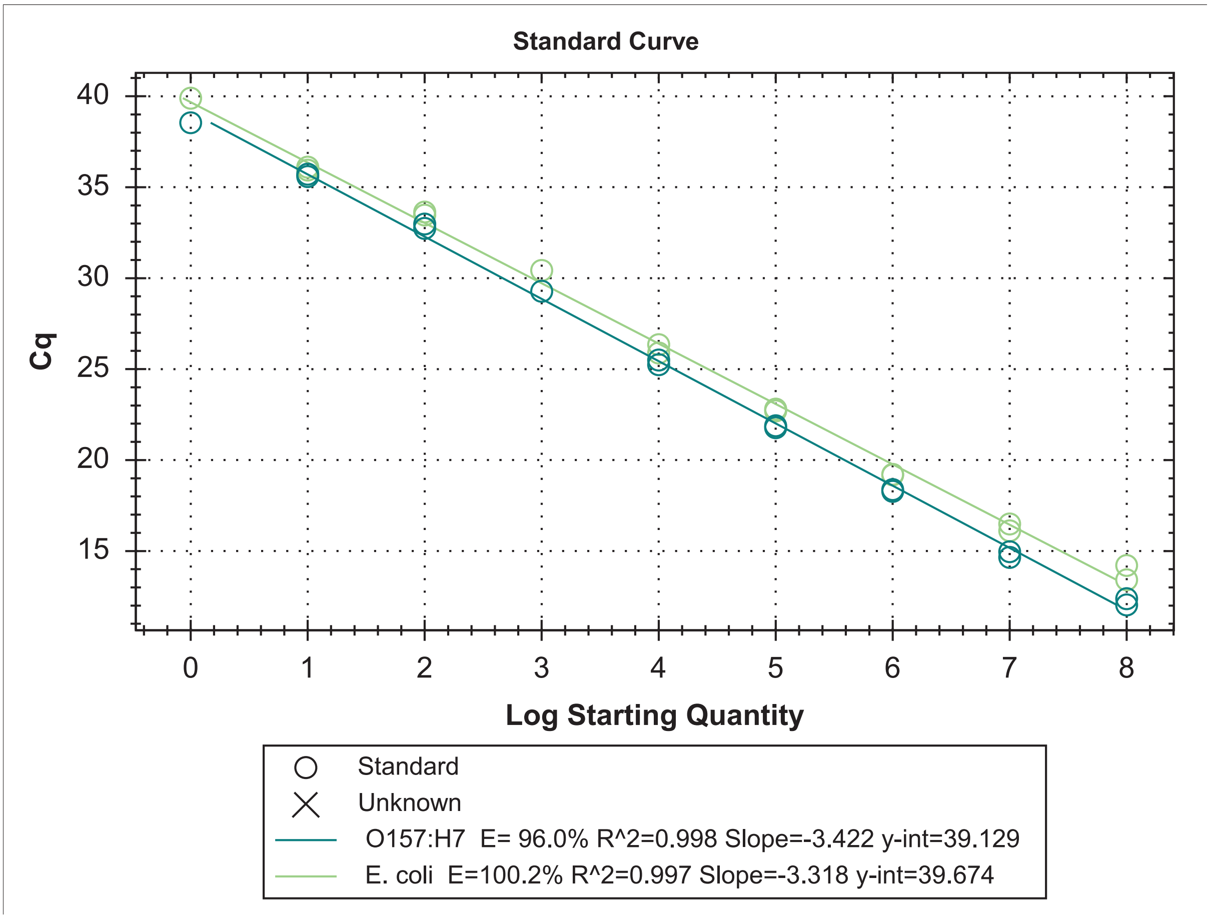

FIGURE S1

Standard curve for the qPCR assay for the detection of E. coli and E. coli O157:H7.

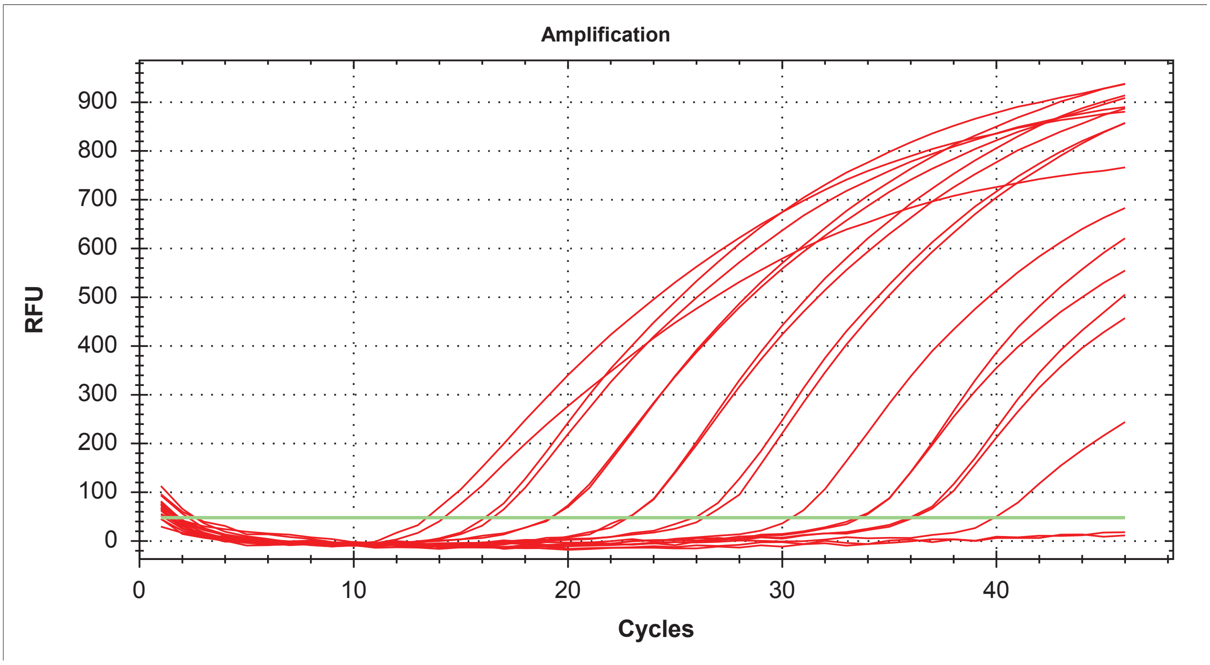

FIGURE S2

Amplification plot for the standard curve of E. coli qPCR assay.

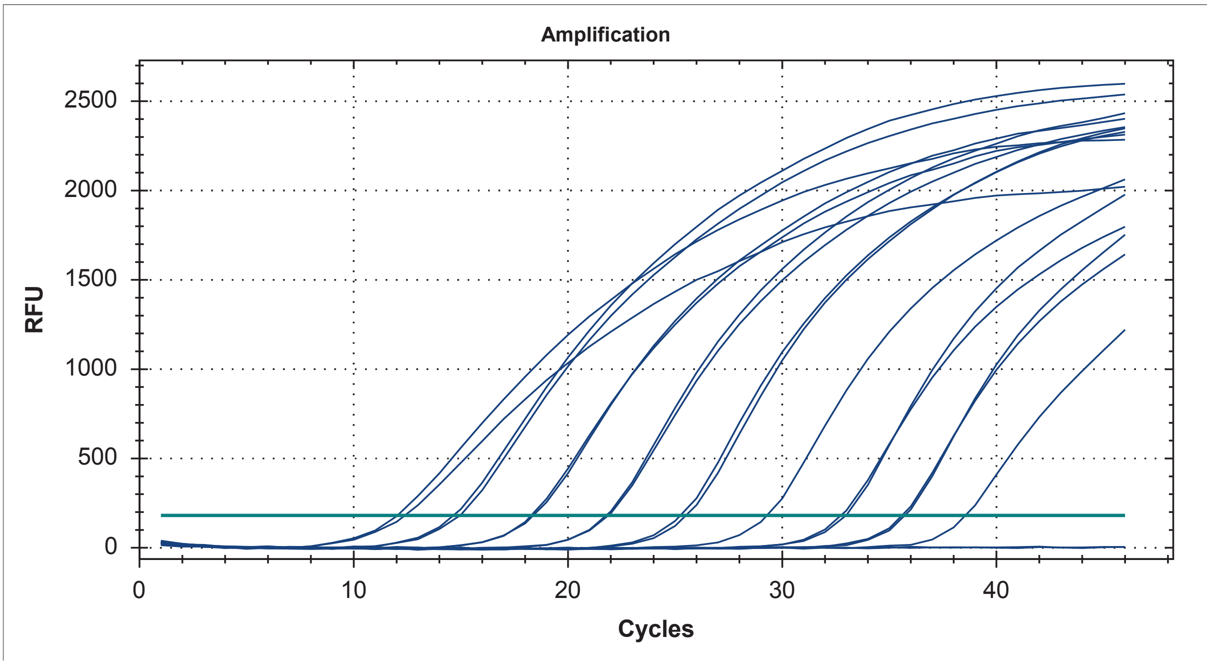

FIGURE S3

Amplification plot for standard curve of E. coli O157:H7 qPCR assay.
